# Pathogenic Characteristics of *Staphylococcus aureus* Endovascular Infection Isolates from Different Clonal Complexes

**DOI:** 10.3389/fmicb.2017.00917

**Published:** 2017-05-19

**Authors:** Dafne Pérez-Montarelo, Esther Viedma, Mercedes Murcia, Irene Muñoz-Gallego, Nieves Larrosa, Patricia Brañas, Nuria Fernández-Hidalgo, Joan Gavaldà, Benito Almirante, Fernando Chaves

**Affiliations:** ^1^Department of Microbiology, Instituto de Investigación Hospital de OctubreMadrid, Spain; ^2^Department of Microbiology, Hospital Universitari Vall d'hebron, Universitat Autónoma de BarcelonaBarcelona, Spain; ^3^Department of Infectious Diseases, Hospital Universitari Vall d'hebron, Universitat Autónoma de BarcelonaBarcelona, Spain

**Keywords:** *Staphylococcus aureus*, clonal complex, virulence, endovascular complications

## Abstract

*Staphylococcus aureus* is a major cause of bacteremia and, even with appropriate clinical management, causes high morbidity, and mortality due to its involvement in endovascular complications and metastatic infections. Through different pathogenic *in vivo* and *in vitro* models we investigated the behavior of *S. aureus* most relevant clonal complexes (CCs) causing endovascular complications. We analyzed 14 *S. aureus* strains representing CC5, CC8, CC15, CC30, and CC45 that caused endovascular complications, including methicillin susceptible and resistant isolates and strains with different functionality of the *agr* global regulator. Their adherence to collagen, interaction with the endothelium, resistance to immune attack, capacity to form biofilm and virulence in the *Galleria mellonella* model were analyzed. CC30 and CC45 showed greater adhesion to collagen and CC8 showed a trend towards higher rate of intracellular persistence in endothelial cells. All CCs exhibited similar tolerance to neutrophil antimicrobial peptide hNP-1 and were capable of forming biofilms under static conditions. The virulence assay in the *G. mellonella* model demonstrated that CC15 and CC30 were the most and least virulent, respectively. The analysis of the genomic sequences of the most relevant virulence genes identified some CC15 specific gene patterns (absence of enterotoxins and *sak* gene) and variants (mainly in leucocidins and proteases), but did not reveal any gene or variant that could be responsible for the increased virulence detected for CC15 strains. Even though all the CCs were capable of causing endovascular complications, our results showed that different CCs are likely to produce these complications through different mechanisms which, if confirmed in more sophisticated models, would indicate the need to more specific management and therapeutic approaches.

## Introduction

*Staphylococcus aureus* is the leading cause of both healthcare- and community-associated bloodstream infections in the industrialized world and is associated with significant morbidity and mortality. *S. aureus* is an opportunistic pathogen that upon entry to the cardiovascular system can lead to serious complications, such as infective endocarditis or thrombophlebitis, resulting in organ failure and death. Previous studies have identified clinical factors that reduce the occurrence of these complications, like early and aggressive antibiotic therapy and removal of intravascular devices (Fowler et al., 2005; Naber, 2009). However, endovascular complications remain commonplace in spite of appropriate management and treatment (Naber, 2009) suggesting that the intrinsic pathogenicity of the *S. aureus* strains involved may play a role in determining clinical outcome and development of endovascular complications.

Although significant progress has been made in the understanding of molecular mechanisms leading to this type of infection investigating specific *S. aureus* genetic markers, much work remains. Moreover, previous reports seem to indicate that no single virulence factor alone is sufficient to describe *S. aureus* endovascular pathogenesis and a cumulative effect from different factors probably offers the most realistic scenario (Peacock et al., 2002; Bouchiat et al., 2015). *S. aureus* has a highly clonal population structure with CCs comprising closely related, although not identical, genetic backgrounds (Lindsay et al., 2006; Dayan et al., 2016). Therefore, the analysis of the pathogenic characteristics of representative *S. aureus* genetic clonal complexes seems an appropriate means by which further our understanding of the etiology of this disease. Indeed, previous studies showed that, although most *S. aureus* genotypes exhibit the capacity to cause invasive disease, bacteremia caused by strains belonging to particular clonal complexes (CC5, CC15, and CC30) has been associated with endovascular complications (Fowler et al., 2007; Nienaber et al., 2011; Bouchiat et al., 2015). Methicillin resistance has also been linked to an increased risk for the development of hematogenous complications (Fowler et al., 2005, 2007). Nevertheless in most cases, the determinants of these associations remain poorly understood.

Several traits have been previously associated with the occurrence of endovascular complications after *S. aureus* bacteremia. An early crucial event in the hematogenous infectious process is the adherence to the endothelium or matrix proteins. Studies in animal models suggested that the ability to interact with collagen, which provides structural support and is present in heart valves, aortic tissues and damaged endothelial tissues, could be advantageous in terms of *S. aureus* endovascular pathogenesis (Patti et al., 1994; Gillaspy et al., 1998). Similarly, the binding of *S. aureus* to endothelial cells, which form the inner lining of cardiovascular system blood vessels, results critical, and leads to impairment of vascular function (Garciarena et al., 2015). Moreover, *S. aureus* can invade and persist intracellularly in endothelial cells, hidden from the host immune system, and extracellular antimicrobial treatment (Proctor et al., 2006; Garzoni and Kelley, 2009; Xiong et al., 2009; Sinha and Fraunholz, 2010). During the initial stages of endovascular pathogenesis, antimicrobial hNP-1 peptide family produced by neutrophils is an important host defense mechanism mediating *S. aureus* clearance (Ganz, 2003; Rigby and DeLeo, 2012). In addition, the ability to form biofilm is an important virulence factor in damaged host tissues (McCarthy et al., 2015).

Therefore, our aim was to characterize the pathogenic behavior of the most relevant *S. aureus* genetic backgrounds causing endovascular complications, using different *in vivo* and *in vitro* models in a collection of representative clinical strains that also allowed us to analyze the effect of methicillin resistance and functionality of the accessory gene regulator (*agr*) that controls virulence factors expression. These strains were analyzed to determine their efficiency of adhesion to collagen; their capacity to adhere to, invade and persist within endothelial cells; their susceptibility to the antimicrobial neutrophil peptide hNP-1; their capacity to form biofilm; as well as their global virulence in the *Galleria mellonella in vivo* model.

## Materials and methods

### Selection of *S. aureus* strains causing endovascular complications

*S. aureus* clinical strains causing endovascular complications were selected from those recovered in two Spanish multicenter studies that focused on complicated catheter-related bacteremia and infective endocarditis (Fernández-Hidalgo, 2016; San-Juan et al., 2016). These studies recorded each patient's clinical history and, most importantly, ensured that their infections were properly managed and treated. The studies were approved by the ethics committee of the Hospital Universitario 12 de Octubre (Madrid, Spain) that considered not necessary to obtain written informed consent because participants were anonymized. A total 259 strains recovered from blood cultures in these studies were processed and isolates were identified according to standard techniques. The strains were characterized for *agr* functionality by measuring δ-hemolysin production as previously reported (Seidl et al., 2011). Genotypic characterization was performed by pulsed-field gel electrophoresis (PFGE) (Chaves et al., 2005), Multilocus Sequence Typing (MLST) (Enright et al., 2000), and DNA microarrays (Alere) (Monecke et al., 2007, 2008).

Following review of the genotypic and phenotypic characteristics of the whole collection, several *S. aureus* strains, that represent the most common CCs in our collection (CC30, CC5, CC45, and CC8), and those CCs previously associated with endovascular complications (CC5, CC15, and CC30), were selected for further analyses. We randomly selected two methicillin susceptible (MSSA) strains of each CC, trying to include strains with different functionality of the *agr* global regulator when possible. Moreover, methicillin resistant isolates (MRSA), belonging to these CCs were also selected if available (there were no CC30 and CC15 MRSA in our collection; two CC5 MRSA isolates were selected according to the microarrays results to represent ST5 and ST125 strains). The main clinical and microbiological characteristics of the total 14 *S. aureus* strains selected are presented in Table 1.

**Table 1:** **Main clinical and microbiological characteristics of the 14 selected *S. aureus* strains**.

**Strain**	**Acquisition**	**Clinical diagnosis**	**Persistent bacteremia**	**Septic complication**	**Severe sepsis**	**In-hospital mortality**	**CC**	**MSSA/MRSA**	**Functional Agr**	**Agr type**
SA80001	CA	Native mitral valve endocarditis	>3 days	Yes (CNS)	No	No	30	MSSA	Yes	3
SA107	HCA	Catheter-related bacteremia	No	Yes (lung)	No	No	30	MSSA	No	3
SA123	HCA	Native mitral valve endocarditis	No	Yes (thrombophlebitis)	Yes	No	45	MSSA	Yes	1
SA520	HCA	Native mitral valve endocarditis	>3 days	–	No	No	45	MSSA	Yes	1
SA170015	HCA	Prosthetic mitral valve endocarditis	No	No	No	No	45	MRSA	Yes	1
SA103	HCA	Native mitral valve endocarditis	No	Yes (thrombophlebitis)	No	No	5	MSSA	Yes	2
SA80004	HCA	Native mitral valve endocarditis	No	No	No	No	5	MSSA	No	2
SA170006	HCA	Native aortic valve endocarditis	>3 days	Yes (liver, spleen, kidney)	No	No	5	MRSA	No	2
SA180009	HCA	Prosthetic aortic valve endocartidis	>7 days	Yes (perivalvular abscess)	No	Yes	5	MRSA	Yes	2
SA10009	CA	Native mitral valve endocarditis	No	No	No	No	15	MSSA	Yes	2
SA10014	CA	Native aortic valve endocarditis	>7 days	Yes (spleen, kidney)	Yes	Yes	15	MSSA	Yes	2
SA180015	CA	Prosthetic aortic & mitral valve endocarditis	No	Yes (perivalvular abscess)	No	No	8	MSSA	No	1
SA190006	CA	Native aortic valve endocarditis	No	No	No	Yes^*^	8	MSSA	Yes	1
SA70002	HCA	Tricuspid endocarditis	>7 days	Yes (spondylodiscitis)	No	No	8	MRSA	No	1

* *Klebsiella pneumoniae pulmonary infection. CC, clonal complex; MSSA, methicillin susceptible S. aureus; MRSA, methicillin resistant S. aureus; Agr, Accesory global regulator; CA, Community-associated; HCA, Healthcare-associated; CNS, central nervous system*.

### Collagen binding assay

The collagen binding assay was performed according to the method described by Waterhouse and Russell (2006). Briefly, plates were coated with 50 μg collagen, incubated overnight at 4°C, washed, blocked for 2 h at 37°C with 3% bovine serum albumin (BSA) in PBS and washed again. One milliliter of a 10^3^ CFU/ml *S. aureus* suspension was added to each well and incubated at 37°C for 2 h. The number of CFU adhered to the collagen was determined after washing by pouring Mueller-Hinton agar and incubating overnight at 37°C. Results were analyzed by comparing the number of CFU recovered to each particular initial inoculum concentration as determined by viable count.

### Adhesion, invasion, and intracellular persistence to endothelial cells

Endothelial cells were harvested from umbilical cord veins and cultured as previously described (Davis et al., 2007). Adhesion, invasion, and intracellular persistence assays were performed according to Richards et al. (2015). Ten milliliters of a 10^9^ CFU/ml *S. aureus* suspension were added to confluent endothelial cells. The inoculum concentration was confirmed for each experiment by serial dilution and plating. For each strain, three plates were incubated at 37°C in 5% CO_2_ for 2 h. To assess invasion and intracellular persistence, the culture medium from two plates was replaced with Endothelial Cell Growth Medium (EGM-2) with 200 μg/ml gentamicin and the plates were returned to the incubator for either a further 3 h (invasion) or 72 h (intracellular persistence). The third plate was used to analyze cellular adhesion.

At the appropriate time points for each test method, endothelial cells were lysed with 1% triton-X-100 and serial dilutions were prepared and plated to determine viable counts. The adherence, invasion and persistence rates for each strain were expressed as the average percentage (±*SD*) of the initial inoculum recovered from at least three independent experiments.

### Susceptibility to human neutrophil peptide 1 (hNP-1)

Each strain's susceptibility to hNP-1 was assayed by exposing 10^5^ CFU/ml to 10 μg/ml of hNP-1 (Biogen Cientifica SL) in a solution containing 10 nM potassium phosphate, pH 7.4, containing 1% BHIB, for 2 h at 37°C and processed for quantitative culture as previously described (Seidl et al., 2011). The results are expressed as the mean percentage (± *SD*) of the initial inoculum that survived exposure to hNP-1. A minimum of three independent assays were performed for each strain.

### Biofilm formation under static conditions

Biofilm formation under static conditions was analyzed as previously described (Seidl et al., 2011). In brief, *S. aureus* was resuspended to a density of 1.5 McFarland, diluted 1:100 in brain heart infusion broth (BHIB) supplemented with 0.2% glucose, incubated for 18 h at 37°C, washed and stained with Safranin. Absorbance was measured at 490 nm and a value >0.5 was considered indicative of biofilm formation (Seidl et al., 2011). Each strain was tested three times and the results are presented in terms of the minimum absorbance ± standard deviation (*SD*).

### Virulence assay in the *Galleria mellonella in vivo* model

The invertebrate *G. mellonella* (Bichosa) infection model was used to study the virulence of the *S. aureus* strains as previously described (Peleg et al., 2009). To ensure that the observations in our study were not due to differences in growth rates, the growth kinetics of each strain were assessed by measuring the change in OD_600_ in broth cultures over 24 h. Fifteen healthy caterpillars of similar size (~200 mg) were used in each assay and 10 μl of a 10^8^ CFU/ml bacterial suspension were injected into the hemocoel of each caterpillar. After injection, caterpillars were incubated at 37°C and the number of dead caterpillars was recorded every 24 h over a period of 5 days. Dead larvae were identified as those melanised and unresponsive to stimuli. Bacterial colony counts were used to confirm all initial *S. aureus* inocula. Three control groups of larvae were included in each experiment, the first received no injection, the second was inoculated only with phosphate buffered saline (PBS) to monitor for killing due to physical trauma and the third was inoculated with a bacterial strain inactivated at 95°C for 10 min, to discard killing by a non-infectious reaction due to *S. aureus* components. Experiments with more than two dead caterpillars in the control groups were aborted and repeated. At least three independent experiments were performed for all strains.

### Genomic comparison of relevant virulence genes

Whole Genome sequencing of all strains was performed using TruSeq DNA PCR free kits (Illumina, CA) according to recommended procedures. Libraries were sequenced on a single run on Illumina MiSeq intrument (150 bp paired-end reads) to generate a coverage of ~100X, with 350 bp of insert size. A total of ~ 1 million reads were obtained on average for each isolate (range 1,560,800–2,372,112). Reads were quality-trimmed (Q30) and reviewed using fastx_trimmer and fastqc software version 0.10.1 (http://www.bioinformatics.babraham.ac.uk/projects/fastqc/), respectively. Resulting reads were assembled into contigs using SPAdes software (version 3.8, http://bioinf.spbau.ru/spades), and their quality was evaluated with QUAST (version 4.3, http://quast.sourceforge.net/quast). The number of contigs for each genome ranged from 52 to 222. The general characteristics of genomes sequencing and assembly are shown in Supplementary Table 1. This Whole Genome Shotgun project has been deposited at GenBank under the accession numbers MKYX00000000, MKYY00000000, MKYZ00000000, MKZA00000000, MKZB00000000, MKZC00000000, MKZD00000000 MKZE00000000, MKZF00000000, MKZG00000000, MKZH00000000, MKZI00000000, MKZJ00000000, and MKZK00000000. Open reading frames (ORFs) were predicted and annotated with the Rapid Annotations using Subsystems Technology (RAST) server (Aziz et al., 2008). This server identified protein-encoding genes, assigned functions to the genes, and allowed us identifying the most relevant virulence genes and their positions. Additionally, VirulenceFinder website (https://cge.cbs.dtu.dk/services/VirulenceFinder/) was also used for virulence genes identification. The genetic sequences of the virulence genes identified were aligned and compared using the Geneious server (geneious 10.0.06) to identify CC15 specific variants through multiple alignments. We defined as CC15 specific variants those variants detected in CC15 strains that were not present in any other strain belonging to a different CC.

### Statistical analyses

Means and standard deviations for a minimum of three independent experiments were calculated and plotted for each assay. Continuous variables are expressed as mean and standard deviation, and univariate comparisons were made with the Student's *t*-test or Mann-Whitney's *U*-test, as appropriate. Categorical parameters are expressed as absolute number and percentage, and univariate comparisons were performed with the *X*^2^ test, or Fisher's exact test, as appropriate. When significant differences were observed, subsequent stratified analyses were performed. For *G. mellonella* survival analysis, larvae mean survival curves were plotted using the Kaplan-Meier method and differences in survival rates between groups were calculated using the log-rank test. All tests were two-tailed, and a *p* < 0.05 was considered statistically significant. Statistical analysis was performed with SPSS statistics software (version 15.0, SPSS, Chicago, IL, USA).

## Results

### CC30 and CC45 strains showed adhesion to collagen

**Figure 1:**
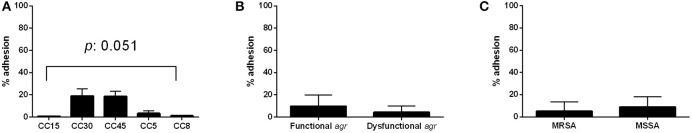
**Adherence to collagen**. Adhesion percentage to collagen according to CC **(A)**, agr functionality **(B)**, and methicillin resistance **(C)**. Bars and error bars represent the mean and standard deviation of at least 3 independent experiments. Only significant *p*-values are shown.

The results obtained for individual strains are shown in Supplementary Figure 1. Comparison of the percentage of bacterial cells bound to collagen between the different CCs showed that CC45 and CC30 had the highest capacity to bind collagen (18.6 and 18.9% inoculum recovery, respectively; Figure 1A) and that CCs 5, 8, and 15 exhibited almost no collagen binding. Significant differences among the different CCs were detected (*p* = 0.051). This could represent an advantage for CC45 and CC30 strains that would adhere easily to the collagen present at endovascular tissues. In contrast, no differences in collagen binding were detected between *agr* functional and dysfunctional strains (9.7 and 4.3%, respectively; *p* = 0.898; Figure 1B) or methicillin resistance phenotype (5.2 and 8.9% for MRSA and MSSA respectively, *p* = 0.5454; Figure 1C).

### CC8 strains presented the highest adhesion, invasion, and intracellular persistence rates to endothelial cells

**Figure 2:**
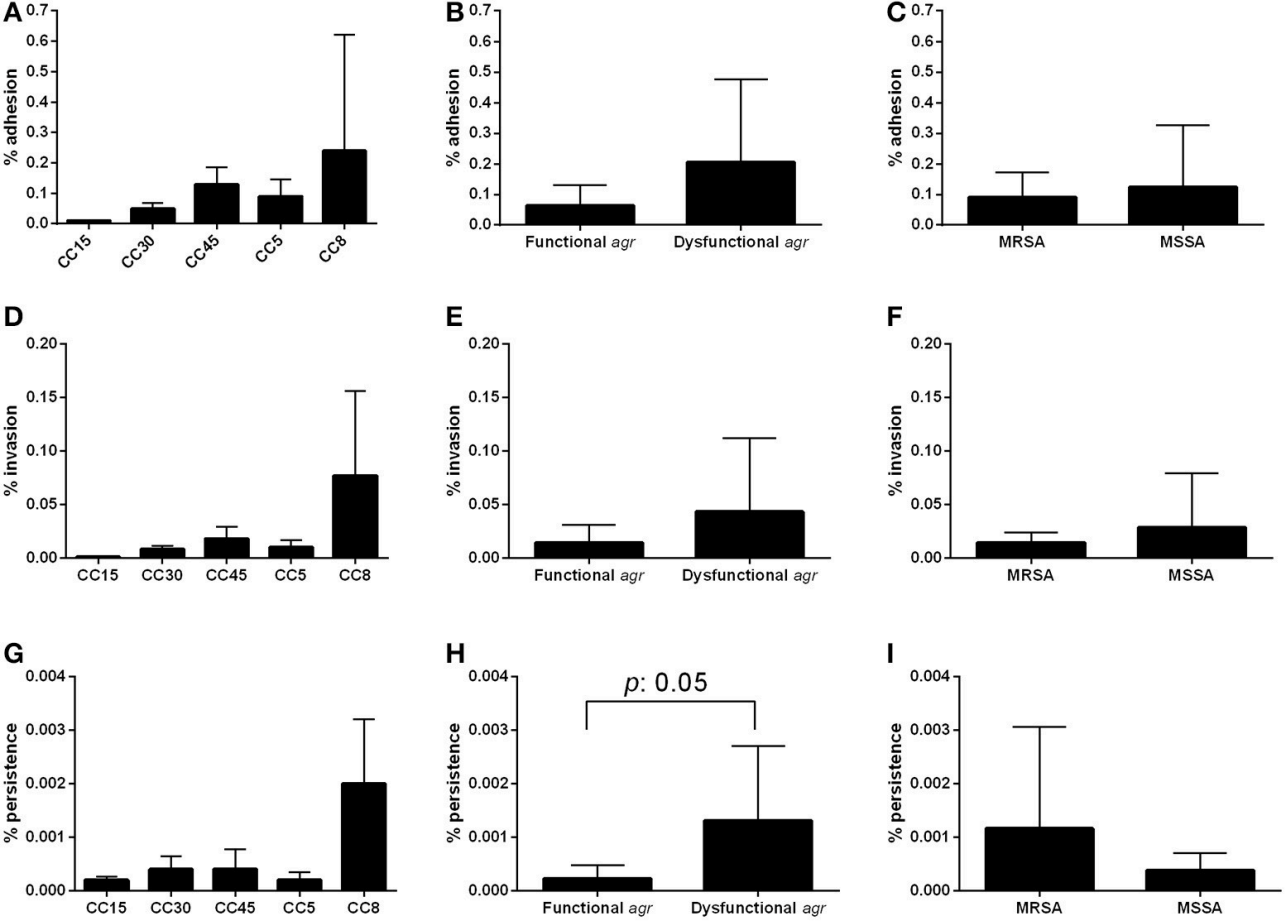
**Adhesion, invasion, and intracellular endothelial cells assay**. Confluent endothelial cell obtained from umbilical cord veins were infected with 10^7^ CFU and the percentage of bacterial cells able to adhere **(A–C)**, invade **(D–F)** and persist intracellularly **(G–I)** is represented according CC, *agr* functionality and methicillin resistance. Results are expressed as the percentage of the initial inoculum recovered. Bars and error bars indicate the mean and standard deviations of at least three independent experiments. Only significant *p*-values are shown.

The results obtained for adhesion, invasion and intracellular persistence of the individual strains are shown in Supplementary Figure 2. The percentage of adhesion to endothelial cells, present at the inner lining of endovascular system vessels, varied among the different CCs and ranged between 0.005% for CC15 to 0.24% for CC8 (Figure 2A). It is necessary to take into account that, among the three CC8 strains included in the study, strain SA180015 showed high levels of adhesion and invasion (results for intracellular persistence were comparable to the other strains), which could distort the overall results for this group for both adhesion and invasion experiments. Additionally, although not statistically significant, a trend toward higher adhesion levels was observed for *agr* dysfunctional strains compared with *agr* functional strains (*p* = 0.518; Figure 2B). No differences in adhesion percentage were observed between strains of MRSA and MSSA (*p* = 0.733; Figure 2C).

A similar pattern was observed among the different CCs for the capacity to invade endothelial cells, with CC15 showing the lowest invasion rate and CC8 the highest, although the differences were not statistically significant (Figure 2D). Indeed, even if we do not consider SA180015, and consider only the other two CC8 strains (SA70002 and SA190006) CC8 is still the one showing the highest invasion percentage. The results observed for invasion when stratified by *agr* functionality and methicillin resistance were also not statistically significant and were comparable to those observed for adhesion (*p* = 0.438 and *p* = 0.454, respectively; Figures 2E,F).

In general, the pattern for intracellular persistence was similar to that observed for adhesion and invasion, being again CC8 strains the ones showing higher intracellular persistence rates, with no statistically significant differences between CCs. Nevertheless, the difference between CC8 and CC5 was almost significant (*p* = 0.056; Figure 2G). Notably, strains with dysfunctional *agr* showed a significantly higher intracellular persistence than strains with functional *agr* (*p* = 0.050; Figure 2H). No significant differences were detected between strains of MRSA and MSSA (*p* = 0.371; Figure 2I). The stratified analysis performed to determine the possible interaction of methicillin resistance on *agr* results showed the same significant trend toward higher intracellular persistence in *agr* dysfunctional strains both in MSSA and MRSA; however results were not significant in either case probably due to the reduced number of strains (*p* = 0.267 and *p* = 0.667, respectively), indicating that *agr* dysfunctional strains were more prone to persist intracellularly regardless of their susceptibility to methicillin. It is worth mentioning that small colony variants (SCV) phenotype was observed in the three CC8 strains and in one CC45 (SA520) and one CC5 (SA80004) in a proportion around 15% in all cases.

### Similar susceptibility to human neutrophil peptide 1 (hNP-1)

**Figure 3:**
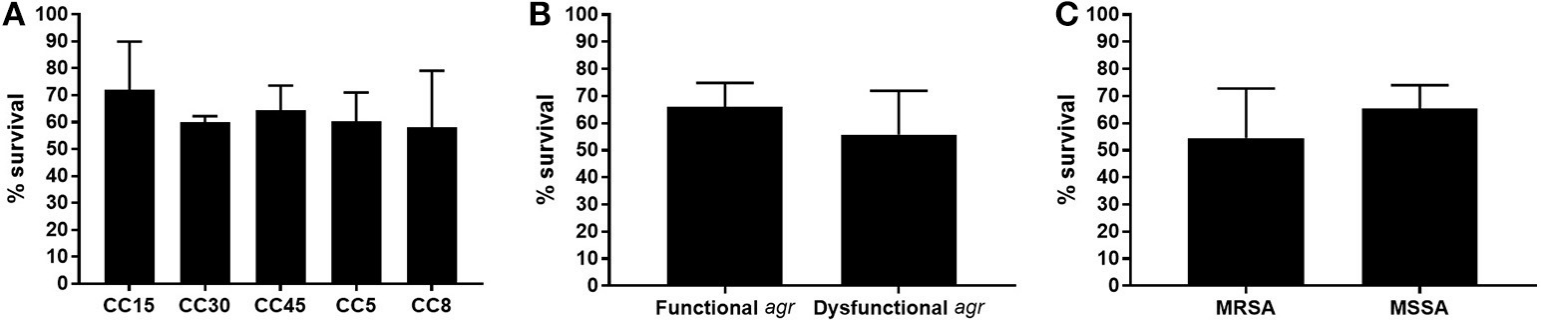
**Susceptibility to hNP-1**. Susceptibility to hNP-1 of the different CCs **(A)**, according to *agr* functionality **(B)**, and methicillin resistance **(C)**. The bars and error bars indicate the means and standard deviations for each group, respectively (at least three independent assays per strain). There are no statistically significant differences.

The results of the susceptibility to hNP-1 obtained for individual strains are shown in Supplementary Figure 3. No significant differences were detected in the percentage of bacterial survival in the presence of hNP-1 peptide between the different CCs, each showing mean survival of around 60% (CC15 72.0%, CC30 60.0%, CC45 64.3%, CC5 60.3%, and CC8 58.1%; *p* = 0.875; Figure 3A). Likewise, no significant differences were detected according to *agr* functionality, with *agr* functional strains exhibiting a mean survival of 65.9% and *agr* dysfunctional strains 55.8% (*p* = 0.364; Figure 3B). Similar bacterial survival was also observed for MRSA and MSSA (54.5 and 65.4%, respectively, *p* = 0.454; Figure 3C).

### All strains formed biofilm under static conditions

**Figure 4:**
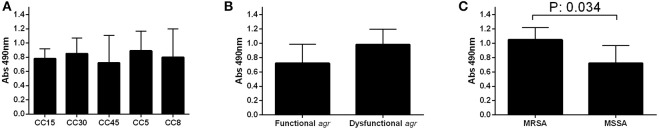
**Biofilm formation**. Biofilm formation of the different CCs **(A)**, according to *agr* functionality **(B)**, and methicillin resistance **(C)**. The bars and error bars indicate the means and standard deviations for each group, respectively (at least three independent assays per strain). Only significant *p*-values are shown.

The biofilm formation of the individual strains are shown in Supplementary Figure 4. The biofilm formation assay showed that clinical isolates from all CCs were capable of forming biofilm under static conditions, with mean absorbance levels of around 0.8 at 490 nm (Figure 4A). No significant differences were detected in the amount of biofilm formed neither between CCs (*p* = 0.926) nor between strains with a functional or dysfunctional *agr*, although it seems to be a trend toward increased absorbance among strains with a dysfunctional *agr* (*p* = 0.089; Figure 4B). Significant differences in the capacity to form biofilm were detected if strains were grouped according to their methicillin resistance phenotype. Strains of MRSA formed more extensive biofilm than those of MSSA with mean absorbance values of 1.05 and 0.72, respectively (*p* = 0.034; Figure 4C). The stratified analysis, performed to determine the possible interaction of *agr* functionality on these results revealed a significant trend toward higher biofilm formation in MRSA than MSSA in agr functional strains (*p* = 0.056), but not in agr dysfunctional strains (*p* = 0.800) indicating that agr functionality is a confounding factor for these results.

### Increased virulence of CC15 strains in the *Galleria mellonella* model

**Figure 5:**
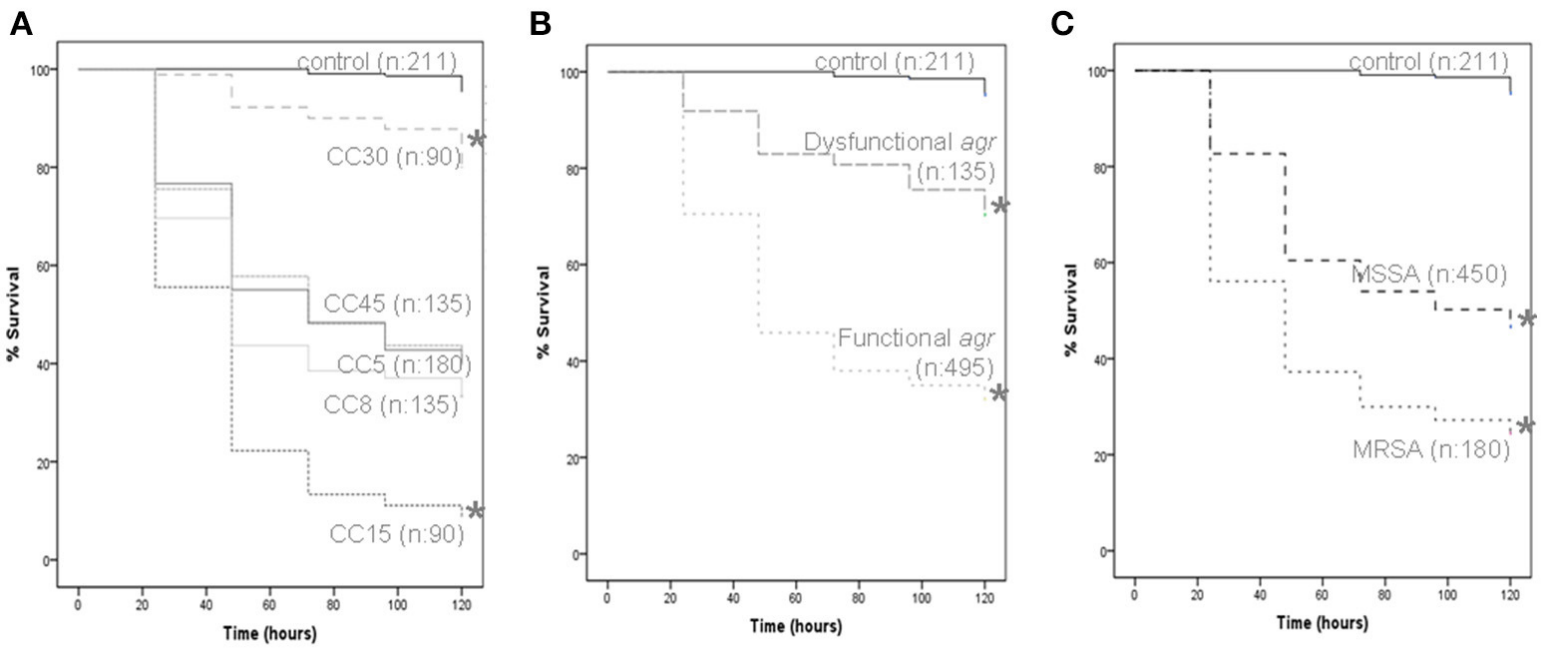
***G. mellonella* virulence assay**. Data from a minimum of three experiments per strain were plotted on Kaplan-Meier survival curves and a log-rank test was used to determine significance. All data grouped according to CC **(A)**, *agr* functionality **(B)**, and methicillin resistance **(C)**. The number of larvae included in each group is indicated (n). Significance is indicated with asterisks, ^*^p < 0.001.

Results from analysis of microbial growth kinetics demonstrated that the growth rates between strains of *S. aureus* were similar (Supplementary Figure 5). However, the *in vivo G. mellonella* model showed differences in virulence between CCs as measured by larvae survival, according to *agr* functionality and methicillin resistance (Figure 5). The results obtained for individual strains in the virulence assay are shown in Supplementary Figure 6. Larvae inoculated with strains from CC30 had the highest mean survival time (112.53 h; *p* < 0.001 for all comparisons), while those inoculated with CC15 had the shortest (48.53 h; *p* < 0.001 for all comparisons), implying an increased virulence of CC15 strains over strains belonging to the other CCs. No significant differences in survival time were demonstrated between larvae infected with CC5 (77.47 h) and CC8 (69.33 h; *p* = 0.14), CC5 and CC45 (78.04 h; *p* = 0.76), and CC8 and CC45 (*p* = 0.1; Figure 5A).

In this model, strains with a functional *agr* regulator were found to be more virulent than those with a dysfunctional *agr* (mean larvae survival time 69.43 and 103.47 h, respectively; *p* < 0.001; Figure 5B). In addition, MRSA strains were found to be more virulent than MSSA strains (mean larvae survival time were 60.13 and 83.36 h, respectively, *p* < 0.001; Figure 5C). For CC5, CC8, and CC45, infection with MRSA resulted in shorter mean larvae survival time than infection with MSSA from the same CC.

### Genomic analysis of the virulence genes present in CC15 strains

In view of the significantly increased virulence of CC15 strains in the *in vivo* model we characterized their repertoire of virulence genes in an attempt to identify genes or variants that could account for this increased pathogenicity. The genomic comparison of the most relevant virulence genes contained in the analyzed strains revealed differences in the content of genes of strains form different CCs. As expected, in general, all strains from the same CC shared the same virulence genes profile, although some differences in particular genes existed (Table 2). There were no specific virulence genes present in both CC15 strains (SA10009 and SA10014) not found in other CCs that could explain the higher virulence detected for these strains in the *in vivo* analysis. Remarkably, strains belonging to CC15 did not contain enterotoxins (except sea/sep in SA10009) which were present in almost all the other strains. Similarly, CC15 strains did not contain the *sak* gene within the Sa3int phage carrying the immune evasion cluster, present in all other strains carrying this phage (all strains except SA80001 and SA70002). The structure of the Sa3int phage, that is located disrupting *hlb* gene, is in all the other strains *scn-chp-sak*, but in the case of CC15 strains it only contained *scn* and *chp* genes. Indeed, when we investigated the DNA microarray results of the whole strains collection we realized that enterotoxins and *sak* genes were absent in mostly all CC15 strains with only a few particular exceptions. The Panton Valentine leukocidin (PVL) encoded by *lukF* and *lukS* genes was present only in SA10009. This leukocidin was only present in this strain in the whole collection so it is not common feature of strains belonging to CC15. It is also worth noting that the *Rot* regulator was not present in SA10014 strain, but as it is not included in the microarray device we could not study its distribution among all the CC15 strains from the collection.

**Table 2:** **Virulence genes distribution of the 14 analyzed strains**.

	**SA80004**	**SA103**	**SA170006**	**SA18009**	**SA180015**	**SA190006**	**SA70002**	**SA10009**	**SA10014**	**SA80001**	**SA107**	**SA123**	**SA520**	**SA170015**
	**CC5**				**CC8**			**CC15**		**CC30**		**CC45**		
**VIRULENCE REGULATORS**														
*Agr*	1	1	1	1	1	1	1	1	1	1	1	1	1	1
*Rot*	1	1	1	1	1	1	1	1	–	1	1	1	1	1
*SarA*	1	1	1	1	1	1	1	1	1	1	1	1	1	1
*CodY*	1	1	1	1	1	1	1	1	1	1	1	1	1	1
*SarR*	1	1	1	1	1	1	1	1	1	1	1	1	1	1
*SaeR*	1	1	1	1	1	1	1	1	1	1	1	1	1	1
*SaeS*	1	1	1	1	1	1	1	1	1	1	1	1	1	1
*SrrA*	1	1	1	1	1	1	1	1	1	1	1	1	1	1
*SigB*	1	1	1	1	1	1	1	1	1	1	1	1	1	1
**ENTEROTOXINS**														
*Seg*	1	1	1	1	–	1	1	–	–	1	1	1	1	1
*Seo*	1	1	1	1	–	1	1	–	–	1	1	1	1	1
*Sen*	1	1	1	1	–	1	1	–	–	1	1	1	1	1
*Sea/sep*	1	1	1	1	–	1	1	1	–	1	1	1	1	1
*Sei*	1	1	1	1	–	1	1	–	–	1	1	1	1	1
*Seu*	1	1	1	1	–	1	1	–	–	1	1	1	1	1
*Sem*	1	1	1	1	–	1	1	–	–	1	1	1	1	1
*Sel*	–	–	–	–	–	–	–	–	–	–	–	1	–	–
*Sec3*	–	–	–	–	–	–	–	–	–	–	–	1	–	–
*Tst*	–	–	–	–	–	–	–	–	–	1	1	–	–	–
**HEMOLYSINS**														
*Hlb*	1^a^	1^a^	1^a^	1^a^	1^a^	1^a^	1^a^	1^a^	1^a^	1	1^a^	1^a^	1^a^	1^a^
*Hla*	1	1	1	1	1	1	1	1	1	1	1	1	1	1
*Hld*	1	1	1	1	1	1	1	1	1	1	1	1	1	1
*hlgB*	1	1	1	1	1	1	1	1	1	1	1	1	1	1
*hlgC*	1	1	1	1	1	1	1	1	1	1	1	1	1	1
*hlgA*	1	1	1	1	1	1	1	1	1	1	1	1	1	1
*Psma1*	1	1	1	1	1	1	1	1	1	1	1	1	1	1
*Psma2*	1	1	1	1	1	1	1	1	1	1	1	1	1	1
*Psma3*	1	1	1	1	1	1	1	1	1	1	1	1	1	1
*Psma4*	1	1	1	1	1	1	1	1	1	1	1	1	1	1
*Psmb1*	1	1	1	1	1	1	1	1	1	1	1	1	1	1
*Psmb2*	1	1	1	1	1	1	1	1	1	–	–	1	1	1
**LEUCOCIDINS**														
*lukE*	1	1	1	1	1	1	1	1	1	–	–	–	–	–
*lukD*	1	1	1	1	1	1	1	1	1	–	–	–	–	–
*lukF*	–	–	–	–	–	–	–	–	–	–	–	–	–	–
*lukS*	–	–	–	–	–	–	–	–	–	–	–	–	–	–
**PROTEASES**														
*Aur*	1	1	1	1	1	1	1	1	1	1	1	1	1	1
*splA*	1	1	1	1	1	1	1	1	1	–	–	–	–	–
*splB*	1	1	1	1	1	1	1	1	1	–	–	–	–	–
*splE*	–	–	–	–	1	1	–	1	1	1	1	–	–	–
**IMMUNE EVASION GENES**														
*Sak*	1	1	1	1	1	1	–	–	–	–	1	1	1	1
*Scn*	1	1	1	1	1	1	–	–	–	–	1	1	1	1
*Chp*	1	1	1	1	1	1	–	–	–	–	1	1	1	1

1 indicates that the gene is present and – that it is absent in each particular strain.

a *Hlb truncated by Sa3int phage*.

The virulence genes sequencing analysis in search of variants found in CC15 strains that were not detected in strains from other CCs showed several CC15 specific mutations, most of them resulting in synonymous single nucleotide substitutions (Table 3). No CC15 specific insertions or deletions were found within these genes. Several variants were detected in the *agr* locus; however this comparison could only be made with CC45 strains that were the only ones with a type II *agr* locus (sequences could not be aligned with the other *agr* types found in the remaining CC5, CC8, and CC30 strains). In general, virulence regulators were highly conserved and we detected only one synonymous mutation at *Rot* regulator (only present in SA10009). A low number of CC15 specific mutations were detected in hemolysin genes with only one non-synonymous mutation in *hla* gene. Several CC15 specific variants were detected in leucocidins and proteases, which resulted the most variable subset of virulence genes, with amino acid changing mutations detected in *lukE, splA*, and *splE*. The *splA* gene showed the highest number of non-synonymous CC15 specific variants with six amino acid changes compared with strains from other CCs. Moreover, two CC15 specific variants, one synonymous and one non-synonymous were identified in the immune evasion gene *chp*.

**Table 3:** **Virulence genes variants especifically detected in CC15 strains**.

	**Total**	**Non-synonymous**	**Synonymous**	**References**
**VIRULENCE REGULATORS**				
AgrB^*^	1		889G>A	DQ157967
AgrD^*^	0	–	–	DQ157967
AgrC^*^	11	–	1739T>C; 1743A>G; 1754G>A; 1763C>T; 1769C>T; 1775A>G; 1787A>T; 1790T>C; 1793T>C; 1796T>C; 2030A>G	DQ157967
AgrA^*^	5	2818A>G (136K>R)	2801C>T; 2810G>A; 2813G>A; 2816T>A	DQ157967
Rot	1	–	126T>A^a^	CP012692
SarA	0	–	–	CP017094
CodY	0	–	–	CP012409
SarR	0	–	–	CP012409
SaeR	0	–	–	CP016861
SaeS	0	–	–	CP016856
SrrA	0	–	–	CP012976
SigB	0	–	–	CP015645
**HEMOLYSINS**				
Hla	1	319C>T (107P>S)^a^	–	LN626917
Hld	0	–	–	DQ157967
HlgB	0	–	–	CP012692
HlgC	1	–	925 G>A	CP012692
HlgA	0	–	–	CP012692
Psma1	0	–	–	CP014444
Psma2	0	–	–	CP014444
Psma3	0	–	–	CP014444
Psma4	0	–	–	CP014444
Psmb1	0	–	–	CP014444
Psmb2	0	–	–	CP014444
**LEUCOCIDINS**				
LukE	3	734G>C (245G>A)^a^	9T>C; 672 G>A	CP012970
LukD	10	–	93 A>G; 189 G>T; 201 T>G; 219 C>T; 234 A>G; 246C>T; 249 A>T; 261A>T; 801 C>T; 921 C>T	CP012970
**PROTEASES**				
Aur	3	–	1187 C/A>G; 1208 A/T>C; 1322A>T	CP010402
SplA	9	10G>A (4G>S); 64A>G (22N>D); 70C>T (24P>S); 257C>T (86S>L); 527A>G (176D>G); 554G>A (185G>E)	231T>C; 438T>C; 618T>C	CP012978
SplB	1	–	495 T>C	CP012978
SplE	1	551C>G (184A>G)	–	CP012978
**IMMUNE EVASION GENES**				
Scn	0	–	–	CP012120
Chp	2	315T>A (46M>L)	256C>T	CP012120

* Results from the comparison of CC15 only with CC45 (type II Agr).

a *Only detected in one of the two CC15 strains*.

## Discussion

In the present study we analyzed the pathogenic characteristics of representative *S. aureus* genetic clonal complexes (e.g., CC5, CC8, CC15, CC30, and CC45) that produced endovascular complications following bacteremia to further our understanding about the genetic background implications on the development of this disease. Our results highlight the relevance of evasion of hNP-1 killing and biofilm formation in the endovascular pathogenesis of *S. aureus* because these traits are shared by all genetic backgrounds analyzed. Nevertheless, we detected important differences in the pathogenic behavior of strains from different CCs regarding their adherence to collagen, capacity to interact with the endothelium and virulence in the *in vivo* model.

Our results indicated that only strains belonging to CC30 and CC45 were able to bind to collagen. This is likely to be due to the presence of the *cna* gene, which is known to encode the primary collagen adhesin, in all the strains analyzed belonging to both CC30 and CC45 genetic backgrounds, but not in the other CCs strains analyzed. Indeed when we revised the DNA microarray data from the whole collection of strains we confirmed that *cna* gene was always present in CC45 and CC30 strains (with only 6 exceptions out of 69 CC30 strains), and it is always absent in CC5, CC8, and CC15 strains (data not shown). The *cna* gene has previously been studied as a *S. aureus* virulence determinant in endocarditis and other infectious diseases models. Results comparing wild type and *cna* mutant strains indicated that cna+ strains caused considerable more septic arthritis symptoms in a mice model and outnumbered the mutant strain 24 h after inoculation in a rat model of infective endocarditis (Patti et al., 1994; Hienz et al., 1996; Nienaber et al., 2011). The results from the present study also suggest that strains that possess this gene, which is associated to particular genetic backgrounds, may take advantage of improved endovascular attachment to establish sites of infection and therefore, further studies in higher models of endovascular complications should be done to further investigate the role of *cna* gene in this pathogenesis.

Our results demonstrated differences in the interaction of the different CCs with endothelial cells, and appear to indicate higher invasion and intracellular persistence among strains of CC8 than among strains belonging to other CCs. The lack of statistical significance in some of these results may be a consequence of the high standard deviation observed among CC8 group. A rate around 15% of SCV was observed in some strains in the intracellular persistence experiment (all CC8, 1 CC5, and 1 CC45) probably caused by the use of gentamicin that induces this phenotype (Massey et al., 2001). We detected differences according to *agr* functionality, with *agr* dysfunctional strains showing greater adhesion, invasive capacity and persistence than strains with a functional *agr*; however these differences were only significant for the intracellular persistence experiment. Moreover, the results from the stratified analysis indicated that *agr* dysfunctional strains were more prone to persist intracellularly, regardless of their susceptibility to methicillin. Fowler et al. (2004) postulated that *agr* dysfunction can foster development of an intracellular reservoir of microorganisms that contributes to the development of infective endocarditis and persistent bacteremia. Moreover, a downregulated and inactive *agr* system has been previously associated to the intracellularly persisting thymidine-dependent SCV phenotype (Kriegeskorte et al., 2014). In general, we did not observe much difference between strains of MRSA and MSSA in terms of their interaction with endothelial cells.

The survival percentages observed in the presence of hNP-1 were relatively high (58–72%) in comparison to previous studies that reported mean survival percentages of 41 and 26% for persistent and resolved bacteremia isolates, respectively (Seidl et al., 2011). Even though we did not detect differences in hNP-1 survival among the different CCs, or according to *agr* functionality or methicillin resistance phenotype, our results demonstrated that strains causing endovascular complications in general exhibit low susceptibility to hNP-1, which may be indicative of increased ability to evade killing by the host's immune defenses.

Our results showed that all the strains we studied were able to form biofilm to a similar extent, with no significant differences among the five CCs. The greater biofilm formation detected in MRSA strains in our study was probably influenced by the distribution of *agr* functionality, because the stratified analysis revealed that these differences were only significant in *agr* functional strains (*p* = 0.056). Moreover, the associated *agr* dysfunction to MRSA strains has been previously reported (Pozzi et al., 2012; McCarthy et al., 2015).

To our knowledge, this is the first report comparing the *in vivo* virulence of different *S. aureus* CCs. The *G. mellonella in vivo* model, has been used to study a wide variety of host-pathogen interactions because there is a correlation between microbial virulence in *G. mellonella* and that in mammals and because it possesses a complex innate immune system (Peleg et al., 2009; Ramarao et al., 2012). The results of our study in this model revealed significant differences in the behavior of the different CCs. Strains from CC30 exhibited low virulence as measured by low larvae mortality. This agrees with previous studies that also reported significant attenuation of virulence for CC30 in both the *G. mellonella* and murine sepsis models (Sharma-Kuinkel et al., 2015). Remarkably, our results indicate high virulence for strains of CC15, with substantial larvae mortality in the first 48 h. Although it has been associated with increased severity of infection and infective endocarditis, there is limited information about the pathogenic mechanisms of CC15 and its involvement in endovascular complications (Fowler et al., 2007; Bouchiat et al., 2015). In agreement with previous reports, we found that strains with a functional *agr*, associated with an up-regulated production of toxins and secreted virulence factors, were significantly more virulent than strains with a dysfunctional *agr* regulator (Peleg et al., 2009). With regard to methicillin resistance, the *G. mellonella* model showed that MRSA strains caused significantly more larvae mortality than strains of MSSA. Previous clinical studies have also demonstrated a significantly higher rate of hematogenous complications with MRSA isolates in catheter-associated bacteremia, suggesting that these strains may harbor virulence determinants not found in clonally related MSSA (Fowler et al., 2005, 2007). Recent reports suggest that, while strains of MRSA that harbor SCCmecII exhibit high PBP2a expression, decreased cytolytic toxin production and *agr* activity, MRSA strains carrying SCCmecIV have variable PBP2a expression and maintain virulence in the absence of antibiotic, trading *agr* activity for methicillin resistance upon exposure to beta-lactam antibiotics (Pozzi et al., 2012; Rudkin et al., 2012). This observation would explain our results because all the analyzed MRSA strains were of the SCCmecIV genotype, and would be expected to maintain virulence in the absence of antimicrobial selective pressure as in the conditions of our experiment. The *G. mellonella* model allowed us to make an initial screening, analyzing 14 strains from five different CCs, and more sophisticated *in vivo* models should be used to validate the most relevant results.

In an effort to identify the genetic determinants that could be responsible for the increased virulence of CC15 strains in the *in vivo* model we characterized their repertoire of virulence genes. The analysis of CC15 virulence genes did not allow us to identify any virulence determinant specifically present in CC15 but not in the other CCs, that could be responsible for the higher virulence caused by these strains. Anyway, it is worth mentioning that CC15 strains showed a particular collection of virulence genes in which enterotoxins and the *sak* immune evasion gene are usually not present. Even though a common characteristic of both CC15 strains explaining their increased virulence could not be detected, the particular strains did present some determinants that could account for this phenotype. In one hand, SA10009 presented the *lukFS* genes coding for PVL, a two component pore-forming exotoxin with an important cytotoxic role on human neutrophils (Löffler et al., 2010), that was not present in any other strain. On the other hand, SA10014 strain did not contain the *Rot* regulator, that represses virulence genes that encode for exo-toxins, hemolysins, proteases and exo-enzymes (Saïd-Salim et al., 2003); therefore its absence could be responsible for an increased virulence. Several CC15 specific variants were detected in the virulence genes analyzed. Overall, leucocidins and proteases showed the highest number of CC15 specific variants. A total of 10 synonymous CC15 specific variants were detected within *lukD* gene and two more within *lukE* (the non-synonymous mutation in *lukE* is present in only one of the CC15 strains). The lukDE is a bi-component pore-forming leukotoxin targeting and killing neutrophils that had been reported as a major virulence factor involved in bloodstream infections that promotes disease progression via its potent cytotoxic effects on phagocytes recruited to hematogenously-seeded infection sites (Alonzo et al., 2012). These authors reported a 100% conservation at the amino acid level in a collection of publicly available sequenced *S. aureus* strains. Even though the detected variants were synonymous and low impact at the protein level is expected, further studies would be needed to discard a potential effect of these variants on virulence. Regarding proteases, *splA* showed the highest number of CC15 specific variants, with a total of six non-synonymous and three synonymous mutations. *SplA* is one of the serin protease-like proteins that had demonstrated to modulate *S. aureus* physiology and virulence in a rabbit model of pneumonia from which authors concluded that *splA* may promote invasion and spreading by removing mucin 16 from epithelial cells, what could facilitate infection at multiple body sites (Paharik et al., 2016). The CC15 specific variants detected do not localized in the catalytic or relevant residues described by Stec-Niemczyk et al. (2009) within *slpA* structure. Therefore, the CC15 *splA* specific amino acid variants identified should be further investigated to elucidate its potential impact in virulence.

Our results indicated that strains from CC30 showed reduced virulence in the *G. mellonella* model and higher binding to collagen, in agreement with previous reports that associated the acquisition of genetic variants of *agrC, hla*, and *psm*α*3* genes in this CC with attenuated virulence in animal models and reduced pro-inflammatory potential (Cheung et al., 2014; Sharma-Kuinkel et al., 2015). Conversely, our results indicate that strains from CC15 are highly virulent in the *G. mellonella in vivo* model, and that this is not due to a greater propensity for biofilm formation, reduced susceptibility to hNP-1, increased capacity to adhere to collagen and endothelial cells, or an enhanced ability to invade and persist intracellularly. The genomic analysis of CC15 virulence genes, even though identified some specific features of CC15 virulence profile, did not allow us to identify any gene that could be responsible for this increased virulence. Moreover, although several CC15 specific variants were detected in these genes, none of them seems to have a direct impact in virulence. Transcriptomic studies would be needed to further investigate the hitherto unknown pathogenic mechanism(s) responsible for the high larvae mortality caused by these strains of CC15.

We recognize some limitations in the present study. The relatively small number of strains affords a substantial chance for statistical bias and does not allow performing proper multivariate analyses; therefore results should be taken cautiously and larger strain cohorts are needed to validate our findings. However, we performed extensive and time-consuming *in vivo* and *in vitro* experiments with all isolates, which cannot be performed automatically at a large scale. In addition, we performed *in vitro* testing of hNP-1 at sublethal concentrations below those that would be encountered *in vivo*. We did not identify the genetic factors or underlying pathogenic mechanism(s) responsible for the different behaviors observed among CCs in the *in vivo* model, and further investigation is required to establish the determinants of these observations. The strengths of the present study include the focus on a specific and well-defined type of infection and outcome that enable drawing firm conclusions. In addition, we selected a representative collection of clinical *S. aureus* strains from multicenter studies within the most relevant and common CCs causing endovascular complications and performed a variety of *in vivo* and *in vitro* experiments to analyze different pathogenic characteristics that might be associated with worse clinical outcome.

## Conclusions

Despite the fact that all the strains included in the study were known to be capable of causing endovascular complications, our results point out differences in their behavior and/or pathogenic potential. This suggests that the development of endovascular complications can occur through different mechanisms such that each particular CC takes advantage of its specific virulence determinants. These may include higher cytotoxicity as observed with CC15 or increased ability to interact with host endothelium as seen with CC30, CC45, and CC8. Our genomic approach identified some particular CC15 specific features, but transcriptomic analysis would be needed to investigate CC15 pathogenic mechanism in detail. The characterization of the behavior of *S. aureus* most relevant clonal complexes (CCs) causing endovascular complications presented in this work is a needed step before proceeding to more sophisticated models to further understand the etiology of this disease. If the differences detected were confirmed in higher models it would help identifying those patients with poor prognosis and indicate the need to recommend more specific management or therapeutic approaches to the different *S. aureus* CCs according their pathogenic potential.

## Author contributions

Conceived and designed the experiments: DP, FC, and BA; Sample analysis: MM, NL, EV, and NF; Performed the experiments: DP, IM, and PB; Analyzed the data: DP; Draft the manuscript: DP, FC, and EV; Revised the manuscript: NL, NF, JG, and BA; Reviewed and approved the final version of the manuscript: DP, EV, MM, IM, NL, PB, NF, JG, BA, and FC.

## Funding

This work was supported by the Health Research Fund (FIS), Department of Health, Spain; Agency for Health Technology Assessment and Research (PI12/01205, PI15/02013, PI12/0179, and PI15/02125) and Instituto de Salud Carlos III, Subdirección General de Redes y Centros de Investigación Cooperativa, Ministerio de Economía y Competitividad, Spanish Network for Research in Infectious Diseases (REIPI RD12/0015 and 0003) and cofunded by the European Regional Development Fund (FEDER).

### Conflict of interest statement

The authors declare that the research was conducted in the absence of any commercial or financial relationships that could be construed as a potential conflict of interest.

## Supplementary material

Supplementary Figure 1 **Adherence to collagen of the 14 analyzed strains individually**.

Supplementary Figure 2 **Adhesion, invasion, and intracellular endothelial cells assay of the 14 analyzed strains individually**.

Supplementary Figure 3 **Susceptibility to hNP-1 of the 14 analyzed strains individually**.

Supplementary Figure 4 **Biofilm formation of the 14 analyzed strains individually**.

Supplementary Figure 5 **Growth kinetics of all selected *S. aureus* strains**.

Supplementary Figure 6 ***G. mellonella* virulence array of the 14 analyzed strains individually**.

Supplementary Table 1 **General characteristics of *S. aureus* genomes sequencing and assembly**.

## References

[B1] Alonzo F. III. Benson M. A. Chen J. Novick R. P. Shopsin B. Torres V. J. (2012). *Staphylococcus aureus* leucocidin ED contributes to systemic infection by targeting neutrophils and promoting bacterial growth *in vivo*. Mol. Microbiol. 83, 423–435. 10.1111/j.1365-2958.2011.07942.x 22142035 PMC3258504

[B2] Aziz R. K. Bartels D. Best A. A. DeJongh M. Disz T. Edwards R. A.. (2008). The RAST Server: rapid annotations using subsystems technology. BMC Genomics 9:75. 10.1186/1471-2164-9-75 18261238 PMC2265698

[B3] Bouchiat C. Moreau K. Devillard S. Rasigade J. P. Mosnier A. Geissmann T.. (2015). *Staphylococcus aureus* infective endocarditis versus bacteremia strains: subtle genetic differences at stake. Infect. Genet. Evol. 36, 524–530. 10.1016/j.meegid.2015.08.029 26318542

[B4] Chaves F. Garcia-Martinez J. de Miguel S. Sanz F. Otero J. R. (2005). Epidemiology and clonality of methicillin-resistant and methicillin-susceptible *Staphylococcus aureus* causing bacteremia in a tertiary-care hospital in Spain. Infect. Control Hosp. Epidemiol. 26, 150–156. 10.1086/502519 15756885

[B5] Cheung G. Y. Kretschmer D. Duong A. C. Yeh A. J. Ho T. V. Chen Y.. (2014). Production of an attenuated phenol-soluble modulin variant unique to the MRSA clonal complex 30 increases severity of bloodstream infection. PLoS Pathog. 10:e1004298. 10.1371/journal.ppat.1004298 25144687 PMC4140855

[B6] Davis J. Crampton S. P. Hughes C. (2007). Isolation of Human Umbilical Vein Endothelial Cells (HUVEC). J. Vis. Exp. 3:e183 10.3791/183 PMC2576276 18978951

[B7] Dayan G. H. Mohamed N. Scully I. L. Cooper D. Begier E. Eiden J.. (2016). *Staphylococcus aureus*: the current state of disease, pathophysiology and strategies for prevention. Expert Rev. Vaccines 15, 1373–1392. 10.1080/14760584.2016.1179583 27118628

[B8] Enright M. C. Day N. P. Davies C. E. Peacock S. J. Spratt B. G. (2000). Multilocus sequence typing for characterization of methicillin-resistant and methicillin-susceptible clones of *Staphylococcus aureus*. J. Clin. Microbiol. 38, 1008–1015. 10698988 10.1128/jcm.38.3.1008-1015.2000 PMC86325

[B9] Fernández-Hidalgo N. Larrosa N. Pérez-Montarelo D. Viedma E. Sáez C. de Alarcón A. (2016). Phenotypic and genotypic risk factors of in-hospital mortality in *Staphylococcus aureus* infective endocarditis. Preliminary results, in 26th European Congress of Clinical Microbiology and Infectious Diseases (Amsterdam).

[B10] Fowler V. G. Jr. Justice A. Moore C. Benjamin D. K. Jr. Woods C. W. Campbell S.. (2005). Risk factors for hematogenous complications of intravascular catheter-associated *Staphylococcus aureus* bacteremia. Clin. Infect. Dis. 40, 695–703. 10.1086/427806 15714415

[B11] Fowler V. G. I. Jr. Nelson C. L. McIntyre L. M. Kreiswirth B. N. Monk A. Archer G. L.. (2007). Potential associations between hematogenous complications and bacterial genotype in *Staphylococcus aureus* infection. J. Infect. Dis. 196, 738–747. 10.1086/520088 17674317

[B12] Fowler V. G. Sakoulas G. McIntyre L. M. Meka V. G. Arbeit R. D. Cabell C. H.. (2004). Persistent bacteremia due to methicillin-resistant *Staphylococcus aureus* infection is associated with agr dysfunction and low-level *in vitro* resistance to thrombin-induced platelet microbicidal protein. J. Infect. Dis. 190, 1140–1149. 10.1086/423145 15319865

[B13] Ganz T. (2003). Defensins: antimicrobial peptides of innate immunity. Nat. Rev. Immunol. 3, 710–720. 10.1038/nri1180 12949495

[B14] Garciarena C. D. McHale T. M. Watkin R. L. Kerrigan S. W. (2015). Coordinated molecular cross-talk between *Staphylococcus aureus*, endothelial cells and platelets in bloodstream infection. Pathogens 4, 869–882. 10.3390/pathogens4040869 26690226 PMC4693168

[B15] Garzoni C. Kelley W. L. (2009). *Staphylococcus aureus*: new evidence for intracellular persistence. Trends Microbiol. 17, 59–65. 10.1016/j.tim.2008.11.005 19208480

[B16] Gillaspy A. F. Lee C. Y. Sau S. Cheung A. L. Smeltzer M. S. (1998). Factors affecting the collagen binding capacity of *Staphylococcus aureus*. Infect. Immun. 66, 3170–3178. 9632582 10.1128/iai.66.7.3170-3178.1998 PMC108329

[B17] Hienz S. A. Schennings T. Heimdahl A. Flock J. I. (1996). Collagen binding of *Staphylococcus aureus* is a virulence factor in experimental endocarditis. J. Infect. Dis. 174, 83–88. 10.1093/infdis/174.1.83 8656018

[B18] Kriegeskorte A. Grubmüller S. Huber C. Kahl B. von Eiff C. Proctor RA.. (2014). *Staphylococcus aureus* small colony variants show common metabolic features in central metabolism irrespective of the underlying auxotrophism. Front. Cell. Infect. Microbiol. 4:141. 10.3389/fcimb.2014.00141 25374845 PMC4204524

[B19] Lindsay J. A. Moore C. E. Day N. P. Peacock S. J. Witney A. A. Stabler R. A.. (2006). Microarrays reveal that each of the ten dominant lineages of *Staphylococcus aureus* has a unique combination of surface-associated and regulatory genes. J. Bacteriol. 188, 669–676. 10.1128/JB.188.2.669-676.2006 16385056 PMC1347281

[B20] Löffler B. Hussain M. Grundmeier M. Brück M. Holzinger D. Varga G.. (2010). *Staphylococcus aureus* panton-valentine leukocidin is a very potent cytotoxic factor for human neutrophils. PLoS Pathog. 6:e1000715. 10.1371/journal.ppat.1000715 20072612 PMC2798753

[B21] Massey R. C. Buckling A. Peacock S. J. (2001). Phenotypic switching of antibiotic resistance circumvents permanent costs in *Staphylococcus aureus*. Curr. Biol. 11, 1810–1814. 10.1016/S0960-9822(01)00507-3 11719226

[B22] McCarthy H. Rudkin J. K. Black N. S. Gallagher L. O'Neill E. O'Gara J. P. (2015). Methicillin resistance and the biofilm phenotype in *Staphylococcus aureus*. Front. Cell. Infect. Microbiol. 5:1. 10.3389/fcimb.2015.00001 25674541 PMC4309206

[B23] Monecke S. Berger-Bachi B. Coombs G. Holmes A. Kay I. Kearns A.. (2007). Comparative genomics and DNA array-based genotyping of pandemic *Staphylococcus aureus* strains encoding panton-valentine leukocidin. Clin. Microbiol. Infect. 13, 236–249. 10.1111/j.1469-0691.2006.01635.x 17391377

[B24] Monecke S. Slickers P. Ehricht R. (2008). Assignment of *Staphylococcus aureus* isolates to clonal complexes based on microarray analysis and pattern recognition. FEMS Immunol. Med. Microbiol. 53, 237–251. 10.1111/j.1574-695X.2008.00426.x 18507678

[B25] Naber C. K. (2009). *Staphylococcus aureus* bacteremia: epidemiology, pathophysiology, and management strategies. Clin. Infect. Dis. 48(Suppl. 4), S231–S237. 10.1086/598189 19374578

[B26] Nienaber J. J. Sharma Kuinkel B. K. Clarke-Pearson M. Lamlertthon S. Park L. Rude T. H.. (2011). Methicillin-susceptible *Staphylococcus aureus* endocarditis isolates are associated with clonal complex 30 genotype and a distinct repertoire of enterotoxins and adhesins. J. Infect. Dis. 204, 704–713. 10.1093/infdis/jir389 21844296 PMC3156104

[B27] Paharik A. E. Salgado-Pabon W. Meyerholz D. K. White M. J. Schlievert P. M. Horswill A. R. (2016). The Spl serine proteases modulate *Staphylococcus aureus* protein production and virulence in a rabbit model of pneumonia. mSphere 12:1 10.1128/mSphere.00208-16 PMC5061998 27747296

[B28] Patti J. M. Bremell T. Krajewska-Pietrasik D. Abdelnour A. Tarkowski A. Rydén C.. (1994). The *Staphylococcus aureus* collagen adhesin is a virulence determinant in experimental septic arthritis. Infect. Immun. 62, 152–161. 8262622 10.1128/iai.62.1.152-161.1994 PMC186080

[B29] Peacock S. J. Moore C. E. Justice A. Kantzanou M. Story L. Mackie K.. (2002). Virulent combinations of adhesin and toxin genes in natural populations of *Staphylococcus aureus*. Infect. Immun. 70, 4987–4996. 10.1128/IAI.70.9.4987-4996.2002 12183545 PMC128268

[B30] Peleg A. Y. Monga D. Pillai S. Mylonakis E. Moellering R. C. Eliopoulos G. M. (2009). Reduced susceptibility to vancomycin influences pathogenicity in *Staphylococcus aureus* infection. J. Infect. Dis. 199, 532–536. 10.1086/596511 19125671 PMC3750955

[B31] Pozzi C. Waters E. M. Rudkin J. K. Schaeffer C. R. Lohan A. J. Tong P.. (2012). Methicillin resistance alters the biofilm phenotype and attenuates virulence in *Staphylococcus aureus* device-associated infections. PLoS Pathog. 8:e1002626. 10.1371/journal.ppat.1002626 22496652 PMC3320603

[B32] Proctor R. A. von Eiff C. Kahl B. C. Becker K. McNamara P. Herrmann M.. (2006). Small colony variants: a pathogenic form of bacteria that facilitates persistent and recurrent infections. Nat. Rev. Microbiol. 4, 295–305. 10.1038/nrmicro1384 16541137

[B33] Ramarao N. Nielsen-Leroux C. Lereclus D. (2012). The insect *Galleria mellonella* as a powerful infection model to investigate bacterial pathogenesis. J. Vis. Exp. 70:e4392 10.3791/4392 PMC3567165 23271509

[B34] Richards R. L. Haigh R. D. Pascoe B. Sheppard S. K. Price F. Jenkins D.. (2015). Persistent *Staphylococcus aureus* isolates from two independent cases of bacteremia display increased bacterial fitness and novel immune evasion phenotypes. Infect. Immun. 83, 3311–3324. 10.1128/IAI.00255-15 26056388 PMC4496624

[B35] Rigby K. M. DeLeo F. R. (2012). Neutrophils in innate host defense against *Staphylococcus aureus* infections. Semin. Immunopathol. 34, 237–259. 10.1007/s00281-011-0295-3 22080185 PMC3271231

[B36] Rudkin J. K. Edwards A. M. Bowden M. G. Brown E. L. Pozzi C. Waters E. M.. (2012). Methicillin resistance reduces the virulence of healthcare-associated methicillin-resistant *Staphylococcus aureus* by interfering with the agr quorum sensing system. J. Infect. Dis. 205, 798–806. 10.1093/infdis/jir845 22301683 PMC3318674

[B37] Saïd-Salim B. Dunman P. M. McAleese F. M. Macapagal D. Murphy E. McNamara P. J.. (2003). Global regulation of *Staphylococcus aureus* genes by Rot. J. Bacteriol. 185, 610–619. 10.1128/JB.185.2.610-619.2003 12511508 PMC145333

[B38] San-Juan R. Viedma E. Chaves F. Lalueza A. Fortún J. Loza E. (2016). High MICs for vancomycin and daptomycin and complicated catheter-related bloodstream infections with methicillin-sensitive *Staphylococcus aureus*. Emerg. Infect. Dis. 6, 1057–1066. 10.3201/eid2206.151709 PMC4880091 27192097

[B39] Seidl K. Chen L. Bayer A. S. Hady W. A. Kreiswirth B. N. Xiong Y. Q. (2011). Relationship of agr expression and function with virulence and vancomycin treatment outcomes in experimental endocarditis due to methicillin-resistant *Staphylococcus aureus*. Antimicrob. Agents Chemother. 55, 5631–5639. 10.1128/AAC.05251-11 21968365 PMC3232782

[B40] Sharma-Kuinkel B. K. Mongodin E. F. Myers J. R. Vore K. L. Canfield G. S. Fraser C. M.. (2015). Potential influence of *Staphylococcus aureus* clonal complex 30 genotype and transcriptome on hematogenous infections. Open Forum Infect. Dis. 2:ofv093. 10.1093/ofid/ofv093 26213692 PMC4512144

[B41] Sinha B. Fraunholz M. (2010). *Staphylococcus aureus* host cell invasion and post-invasion events. Int. J. Med. Microbiol. 300, 170–175. 10.1016/j.ijmm.2009.08.019 19781990

[B42] Stec-Niemczyk J. Pustelny K. Kisielewska M. Bista M. Boulware K. T. Stennicke H. R.. (2009). Structural and functional characterization of SplA, an exclusively specific protease of *Staphylococcus aureus*. Biochem. J. 419, 555–564. 10.1042/BJ20081351 19175361

[B43] Waterhouse J. C. Russell R. R. (2006). Dispensable genes and foreign DNA in *Streptococcus mutans*. Microbiology 152(Pt 6), 1777–1788. 10.1099/mic.0.28647-0 16735740

[B44] Xiong Y. Q. Fowler V. G. Yeaman M. R. Perdreau-Remington F. Kreiswirth B. N. Bayer A. S. (2009). Phenotypic and genotypic characteristics of persistent methicillin-resistant *Staphylococcus aureus* bacteremia *in vitro* and in an experimental endocarditis model. J. Infect. Dis. 199, 201–208. 10.1086/595738 19086913 PMC2827482

