# Supplementary figures and images for: Pathogenic Characteristics of Staphylococcus aureus Endovascular Infection Isolates from Different Clonal Complexes
Source: Front Microbiol. 2017 May 19;8:917. doi: 10.3389/fmicb.2017.00917 (PMC5437158; doi:10.3389/fmicb.2017.00917)

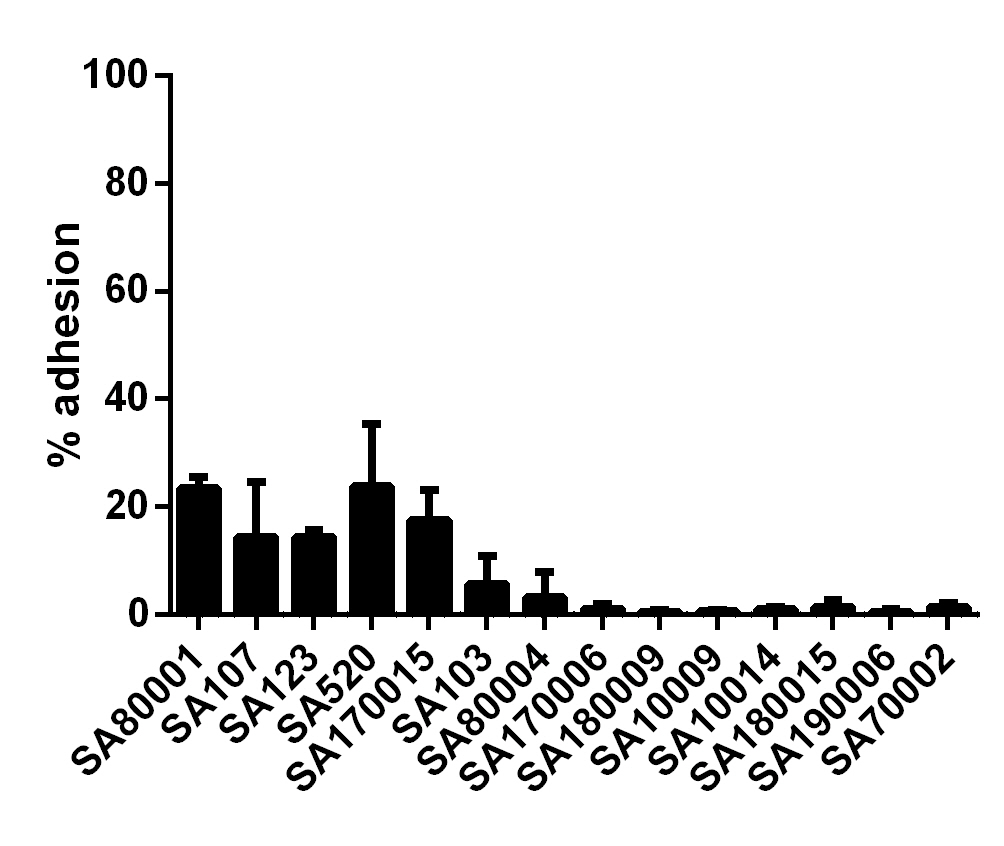

Supplement: Supplementary Figure 1 — Adherence to collagen of the 14 analyzed strains individually. [file SupplementaryFigure1.JPEG]

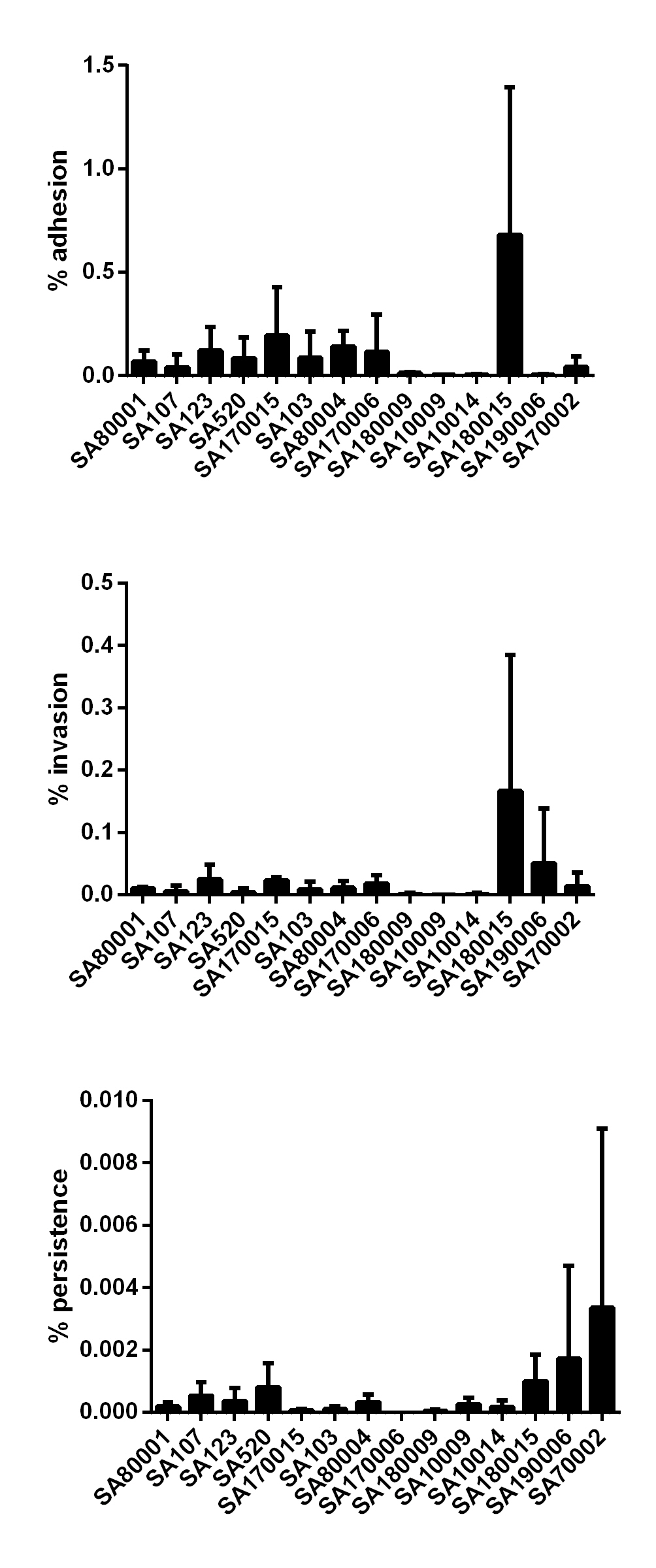

Supplement: Supplementary Figure 2 — Adhesion, invasion, and intracellular endothelial cells assay of the 14 analyzed strains individually. [file SupplementaryFigure2.JPEG]

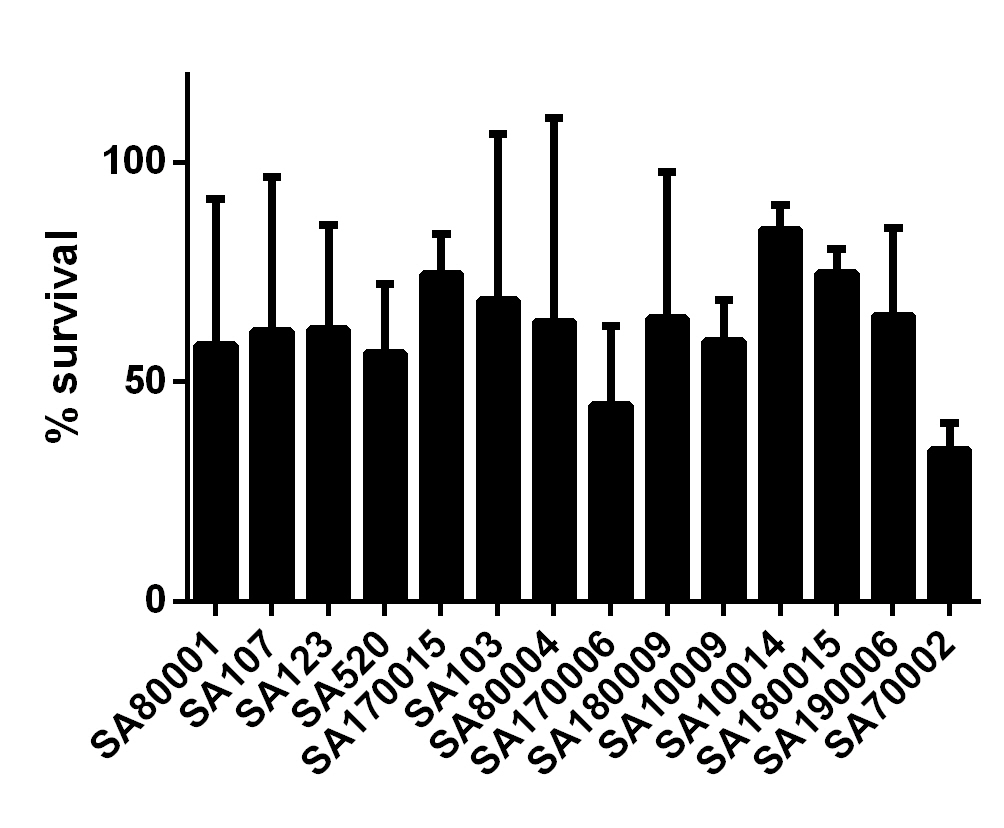

Supplement: Supplementary Figure 3 — Susceptibility to hNP-1 of the 14 analyzed strains individually. [file SupplementaryFigure3.JPEG]

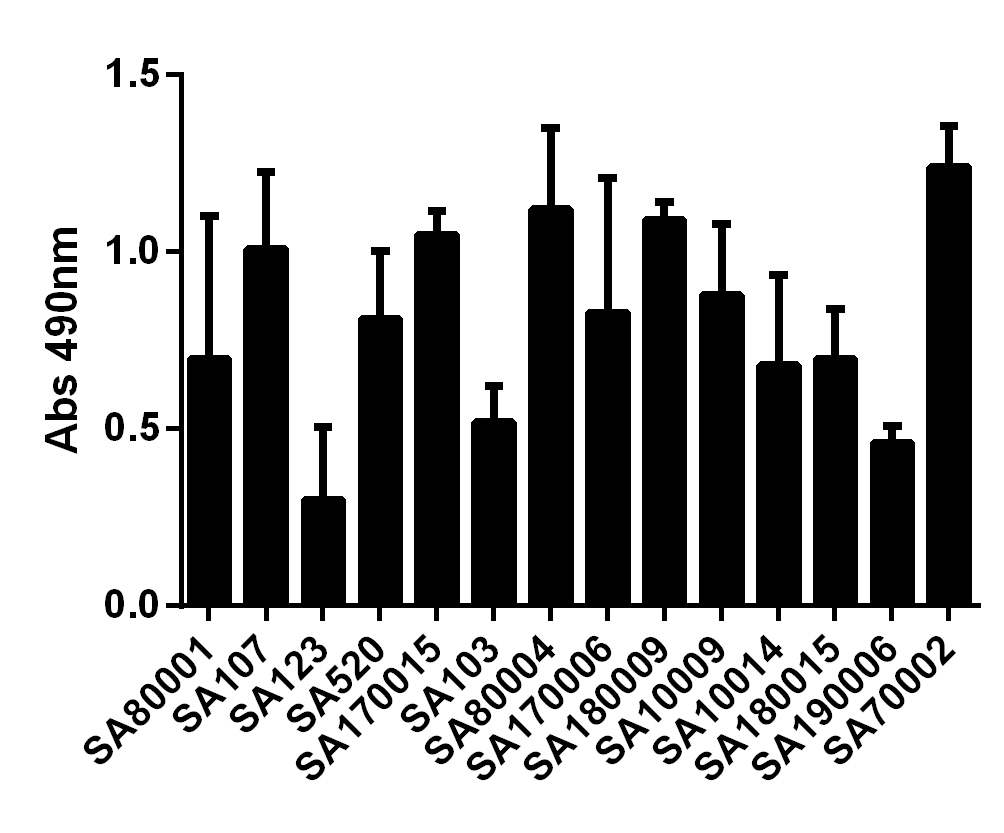

Supplement: Supplementary Figure 4 — Biofilm formation of the 14 analyzed strains individually. [file SupplementaryFigure4.JPEG]

**Supplementary Figure 5:** Growth kinetics of all selected *S. aureus* strains.

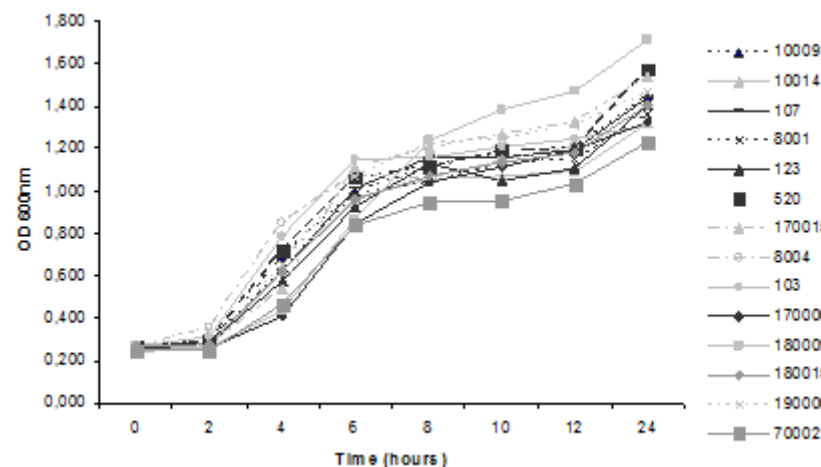

Supplement: Supplementary Figure 5 — Growth kinetics of all selected S. aureus strains. [file SupplementaryFigure5.PDF]

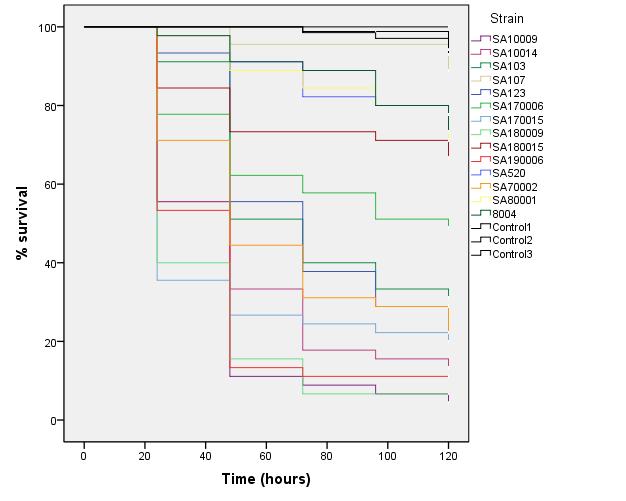

Supplement: Supplementary Figure 6 — G. mellonella virulence array of the 14 analyzed strains individually. [file SupplementaryFigure6.JPEG]
