# Supplementary material for: Pathogenic Characteristics of Staphylococcus aureus Endovascular Infection Isolates from Different Clonal Complexes
Source: Front Microbiol. 2017 May 19;8:917. doi: 10.3389/fmicb.2017.00917 (PMC5437158; doi:10.3389/fmicb.2017.00917)
Supplement: Supplementary Table 1 — General characteristics of S. aureus genomes sequencing and assembly. [file Table1.PDF]

Supplementary Table 1. General characteristics of *S. aureus* genomes sequencing and assembly.

	Whole genome sequencing Illumina MiSeq			Assembly statistics					Annotation		
Strain	Reads (bp)	Q30	k-mer	Size (bp)	Contigs (nº)	Contigs >500 bp (nº)	N50	L50	G+C content (%)	Protein coding genes (nº)	Transfer RNA genes (nº)
SA80001	2,284,184	95.580	K-77	2,758,524	96	56	134,523	6	32,7	2,581	69
SA107	2,265,758	95.857	K-77	2,835,164	125	68	175,176	5	32,9	2,648	74
SA123	2,372,112	94.780	K-77	2,712,951	52	14	479,394	2	32,8	2,519	74
SA520	1,560,800	95.059	K-55	2,702,993	88	25	184,958	5	32,8	2,491	57
SA170015	2,022,364	94.246	K-77	2,753,028	55	17	428,903	3	32,8	2566	77
SA80004	2,269,552	95.077	K-77	2,767,789	55	19	369,340	4	32,7	2,575	76
SA103	2,069,484	95.394	K-77	2,785,061	65	35	379,223	3	32,8	2,615	76
SA170006	2,350,848	94.048	K-77	2,942,833	222	112	178,786	5	32,9	2739	73
SA180009	1,954,570	93.567	K-77	2,800,935	56	27	243,848	4	32,7	2614	75
SA10009	1,950,602	94.847	K-77	2,754,921	81	26	192,628	5	32,7	2,556	69
SA10014	2,091,466	91.089	K-77	2,503,430	78	23	249,734	4	32,7	2,497	64
SA180015	1,874,410	95.038	K-55	2,762,468	105	33	279251	4	32,7	2,564	46
SA190006	2,009,170	95.784	K-77	2,752,902	115	40	308,536	4	32,9	2,556	78
SA70002	2,105,298	95.577	K-77	2,683,166	60	22	290,256	4	32,7	2455	
